# Depicting multiple sclerosis disease course using lesion parenchymal fraction: a quantified expression of the topographical model of multiple sclerosis

**DOI:** 10.1093/braincomms/fcaf280

**Published:** 2025-07-22

**Authors:** Stephen Krieger, Thibo Billiet, Nuno Pedrosa de Barros, Thanh Vân Phan, Wim Van Hecke, Annemie Ribbens, Karin Cook, Tim Wang, Kain Kyle, Linda Ly, Justin Garber, Michael Barnett

**Affiliations:** Department of Neurology, Icahn School of Medicine at Mount Sinai, New York, NY 10029, USA; icometrix, 3012 Leuven, Belgium; icometrix, 3012 Leuven, Belgium; icometrix, 3012 Leuven, Belgium; icometrix, 3012 Leuven, Belgium; icometrix, 3012 Leuven, Belgium; WPP/VML Health, New York, NY 10003, USA; Brain and Mind Centre, University of Sydney, NSW 2050, Sydney, Australia; Sydney Neuroimaging Analysis Centre, NSW 2050, Sydney, Australia; Brain and Mind Centre, University of Sydney, NSW 2050, Sydney, Australia; Sydney Neuroimaging Analysis Centre, NSW 2050, Sydney, Australia; Brain and Mind Centre, University of Sydney, NSW 2050, Sydney, Australia; Sydney Neuroimaging Analysis Centre, NSW 2050, Sydney, Australia; Brain and Mind Centre, University of Sydney, NSW 2050, Sydney, Australia; Department of Neurology, Westmead Hospital, NSW 2145, Australia; Brain and Mind Centre, University of Sydney, NSW 2050, Sydney, Australia; Department of Neurology, Westmead Hospital, NSW 2145, Australia; Department of Neurology, Royal Prince Alfred Hospital, NSW 2050, Sydney, Australia

**Keywords:** multiple sclerosis, MRI, topography, progression, spinal cord

## Abstract

The topographical model of multiple sclerosis proposes that functional reserve compensates for multiple sclerosis lesions, and that disability accumulation is the result of an insidious unmasking of deficits referable to lesion burden. To utilize topographical principles to establish an imaging metric—lesion parenchymal fraction (LPF)—defined as lesion volume divided by parenchymal volume in the same regional compartment, and to assess the relationship of LPF with disability. One hundred patients with relapsing-remitting or secondary progressive multiple sclerosis were evaluated; clinical and MRI data were longitudinally acquired from 2011–19. Lesion and parenchymal volumes in brain and cervical cord were processed using icobrain/icospine pathways, parsed into topographical compartments, and regional LPF was computed. Performance of LPF-based linear models was evaluated using Pearson correlation and root mean squared error between measured and estimated disability scores (expanded disability status score). To establish relative contributions of LPF in cervical, infratentorial and cerebral compartments, a density plot of weight distributions was generated. Individual disability scores and compartment-weighted LPF trajectories were rendered using matplotlib in Python. MRI and clinical data from 78 patients were sufficient for modelling: 39 remaining relapsing-remitting and 39 progressing to secondary progressive multiple sclerosis. The LPF model had the best performance in decoding the disability score using root mean squared error (1.638) and ranked second in Pearson correlation (0.275). Setting the mean coefficient of cerebral LPF to 1, the cervical compartment had the largest coefficient (3.8), followed by infratentorial (2.5). Compartment-weighted cumulative LPF values depict multiple sclerosis disease trajectory longitudinally on a per-patient basis. This visualization is shown for patients who transitioned from relapsing-remitting to secondary progressive phenotypes; non-progressing patients; and outliers where the LPF model does not approximate the disability score trajectory. We developed and evaluated LPF as an MRI-quantified expression of the topographical model of multiple sclerosis, a first effort to operationalize this model to depict individual disease course. That LPF from spinal cord and infratentorial compartments conferred respectively 3.8 and 2.5 more impact on the expanded disability status score than the cerebral hemispheres emphasizes the importance of lesion topography. Implications of outliers are instructive regarding current model limitations; refinement using additional clinical and imaging metrics could allow application to individual patients.

## Teaser

Using a longitudinal multiple sclerosis cohort, Krieger *et al*. established a new metric—lesion parenchymal fraction—defined as lesion volume divided by parenchymal volume in the same regional compartment. The authors utilize this MRI-quantified expression of the topographical model of multiple sclerosis to create novel depictions of individual disease course.

## Graphical Abstract

**Graphical Abstract:**
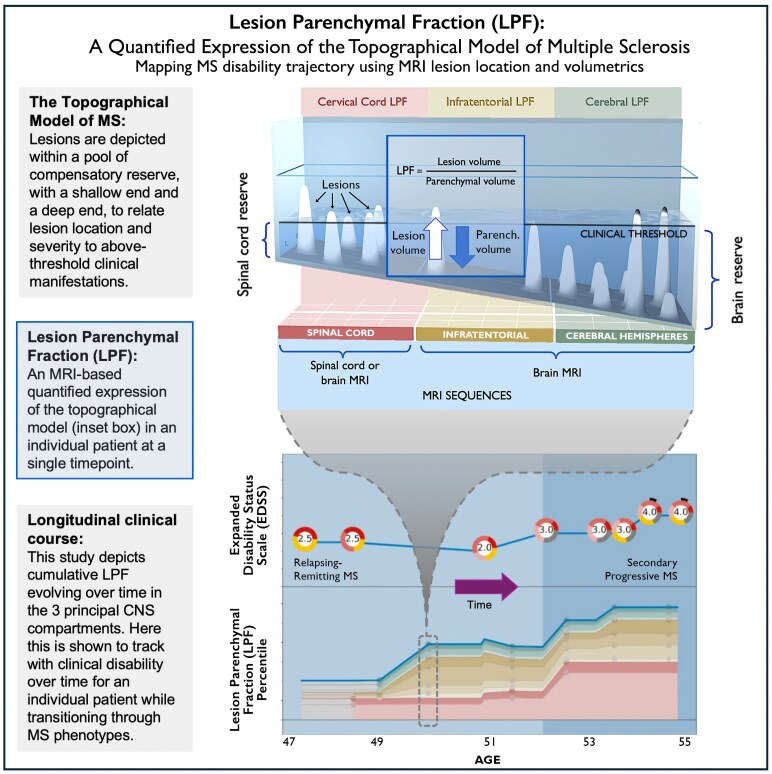

## Introduction

Despite advances in multiple sclerosis (MS), there remain challenges in assessing the burden of disease, particularly at the individual level. One fundamental concept that can inform MS prognosis is that symptomatology maps to lesion location,^1^ and damage to specific regions, such as the brainstem^2^ and spinal cord,^3-7^ has been shown to yield worse disability outcomes including the transition from relapsing-remitting (RRMS) to secondary progressive MS (SPMS).^8^ On a group level, lesion localization is recognized as a prognostically important factor for the likelihood of progressive disability accumulation, including the spatial localization of demyelinating lesions^9^ and even the role of a singular critically-located lesion, often in the upper cervical cord,^1,10,11^ or cervico-medullary junction.^12^ Identifying specific patterns of disability accrual and localization in individuals may allow clinicians to characterize the disease in a more nuanced manner than by clinical phenotype category alone.

Conventional MRI often reveals an extent of lesional disease that is not clinically apparent. This so-called ‘clinical-radiologic paradox’ may be explained partly on the basis that brain lesion burden fails to correlate with disability because MS lesions in the spinal cord, rather than the brain, are the principal cause of motor impairment prioritized by the expanded disability status score (EDSS).^13^ Additionally, compensatory reserve is increasingly understood to mitigate MS severity. It is hypothesized that an accelerated loss of brain volume leads to the loss of neurological reserve, and that progression may become clinically apparent after reserve is depleted.^14^ This concept has been extended into the spinal cord, where the loss of cord reserve^15^ has been shown to have prognostic implications for the development of disability and the conversion to SPMS.^8,16,17^ Extracting more clinically-informative information from conventional imaging and mapping disability accrual to the regions that are most likely to drive progression may help resolve the clinical-radiologic paradox and reveal an important missing piece in our ability to model and predict disease course at an individual level.^1^

**Figure 1:**
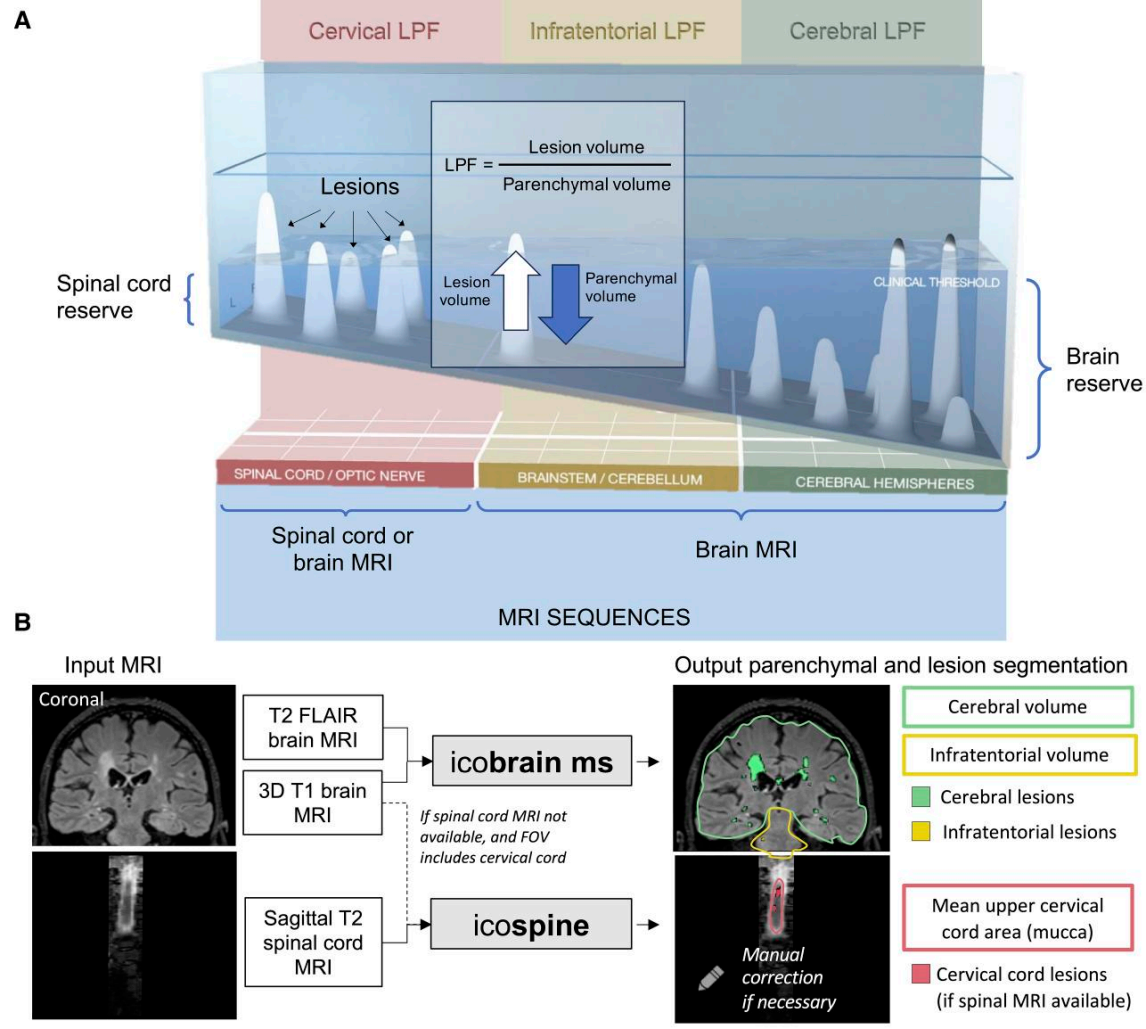
**The topographical model of MS and the lesion parenchymal fraction (LPF) framework.** (**A**) The topographical model conceptualizes the central nervous system (CNS) as a pool of reserve with increasing levels of depth, with the spinal cord at the shallow end, the brainstem and cerebellum with intermediate depth and the cerebral hemispheres comprising the deep end. Focal T2 lesions are represented as topographical peaks that rise up from the pool base. The topographical distribution drives the clinical picture for an individual patient, with functional system deficits referable to lesion localization. The single view shown represents an individual patient’s disease at a single point in time; the water is translucent, with both above-threshold clinical findings and subthreshold lesions shown. The combined volume of above-threshold topographical peaks corresponds with the degree of accumulated disability. Progression is depicted as these peaks becoming increasingly unmasked as reserve declines across CNS compartments, with brain reserve noted at right and spinal cord reserve noted at left. In the inset box, lesion parenchymal fraction (LPF) is defined as a ratio of lesion volume divided by regional parenchymal volume, serving as a quantified measure of the topographical model principles. LPF rises when lesion volume increases or as parenchymal volume declines. LPF is derived from MRI sequences using regional lesion and parenchymal volumes in the three principal topographical model compartments: cervical cord (in red), infratentorial (in yellow) and cerebral hemispheres (in green). (**B**) Imaging method for lesion and regional parenchymal measurement from MRI. Brain MRI input was processed by icobrain software for brain lesion and parenchymal segmentations. Spinal cord MRI and brain MRI with cervical cord were processed by icospine software for cervical cord lesion and parenchymal segmentations. Visual representation of segmented outputs shows parenchymal volume outlines, and individual lesions, colour-coded according to the three topographical model regional compartments.

It was on this basis that the topographical model of MS was proposed as a unifying depiction of MS disease course. This model has been described in detail previously,^18-20^ and will be outlined briefly here as it serves as the underlying concept for this project. The topographical model of MS proposes that functional reserve compensates for focal lesions, and that disability accumulation is the result of an insidious unmasking of deficits referable lesion burden (see Fig. 1A).^18^ The model depicts a pool of compensatory reserve, where the depth of the water corresponds with the degree of reserve intrinsic to these different regions: from the least redundancy in the spinal cord at the ‘shallow end’, to the greatest such structural and functional reserve in the cerebral hemispheres at the ‘deep end’.^18,20^ In this way, the model can be individualized to encapsulate a particular patient’s ‘disease topography’—the individual clinical pattern and severity of disease, with progression dynamically revealed as reserve declines and unmasks clinical signs and symptoms above the clinical threshold.^19^

Here, for the first time, we investigate principles of the topographical model utilizing clinical and MRI data from a well-characterized retrospective cohort. To be most informative, the cohort was selected to include both stable (non-progressing) and progressing patients, with longitudinal follow-up including sequential EDSS and MRI (including cervical cord imaging) for quantitative analysis. While both brain and lesion volume have been shown to have significant correlations with disability status,^21-24^ what has not been done previously is to integrate parenchymal volume and lesion localization together towards a model that can begin to decode disability accumulation on an individual level.

We establish a new MRI-based metric—lesion parenchymal fraction (LPF)—defined as regional lesion volume divided by regional parenchymal volume in the same compartmental localization. This metric serves as a first quantified expression of the factors depicted in the topographical model (see Fig. 1 inset). We evaluate the interaction of regional lesion load and tissue loss and assess if LPF in the three main topographical compartments (spinal cord, infratentorial and cerebral hemispheres) can describe the disability status as measured by the expanded disability status scale (EDSS). We tested if LPF is a better descriptor of EDSS than the parenchymal volume and lesion volumes considered separately, and if a model including both brain and spinal cord MRI better describes EDSS than a model solely based on brain MRI.

To test the assumption that LPF in the shallow end of the reserve pool would preferentially contribute to above-threshold clinical manifestations, we determined the relative weight of LPF for each of the three principal compartments towards estimating EDSS. We then used these compartment-weighted LPF values to create a novel visualization of MS disease burden depicting cumulative LPF and clinical course longitudinally on a per-patient basis. We discuss the extent to which the trajectory of LPF over time matches the severity and clinical pattern of disability for each patient by EDSS and its component functional system scores (FSSs) and consider instructive examples where a patient’s clinical course departs from that which is predicted by our model to garner insights for further refinement.

## Materials and methods

Patients with MS were recruited and clinically evaluated at the Brain and Mind Centre in the University of Sydney, Australia, from 1 January 2011 through 31 December 2019. We refer to these patients as the Sydney Cohort. MRI scans were obtained from different scanner models, with the vast majority being acquired on the three following models: GE MR750 3T (GE Healthcare, Milwaukee, USA), Philips Ingenia 3T (Philips Inc, Amsterdam, The Netherlands) and Siemens Skyra 3T (SIEMENS Healthineers, Erlangen, Germany). Brain MRI and spinal cord MRI were acquired at several time points on a subset of the Sydney Cohort.

### Standard protocol approvals, registrations and patient consents

Written informed consent was obtained from all participants according to the Declaration of Helsinki, and the studies were approved by the local institutional review boards.

### Patients

One hundred patients diagnosed with relapsing-remitting (RRMS) or secondary progressive MS (SPMS) were selected retrospectively from the Sydney Cohort.

Inclusion criteria were: (i) MS phenotype being RRMS at baseline; (ii) age of 18 years or older; and (iii) at least five available brain and/or spinal cord MRI scans acquired over the course of five or more years of follow-up, along with five or more years of clinical follow-up for assessing disability (with EDSS and FSS). Patients with competing/superimposed neurological diagnoses potentially impacting clinical disability measures were excluded during the pre-screening phase. As this study evaluates the transition from RRMS to SPMS, patients with primary progressive MS were excluded.

Available MRI sequences necessary for inclusion were 3D T1 and 3D FLAIR for brain MRI; T2 for cervical lesions, and T2 or 3D T1 sequences for volumetric analyses of spinal cord MRI.

Among the 100 patients included in this retrospective study, 50 patients remained in the RRMS phenotype (‘non-progressing’), and 50 transitioned from RRMS to SPMS (‘progressing’) during the observation period from 2011 through 2019. As in clinical practice, the transition to SPMS was defined retrospectively based on progression in disability scores in the absence of relapse activity. The majority of patients who progressed (42/50) met the MSBASE-defined criteria outlined in Lorscheider *et al*.,^25^ which requires an EDSS > 4; however, to avoid restricting the SPMS category to those with purely ambulatory progression, patients were classified as SPMS if they progressed by at least two steps in other functional system scores in the absence of relapse activity. We included an equal number of patients who had met and not met a standardized definition of SPMS in order to study our model in a population that represented both phenotype categories. The date of onset of progression to SPMS was then taken as the date of the first increase in FSS or EDSS in the absence of relapse. Table 1 summarizes the demographic information for the full cohort, and for the cohort utilized for modelling that included sufficient imaging and clinical variables at unified time points for analysis. A flowchart illustrating how the final sample size was formed can be found in Supplementary Fig. 1 and demographic details of the excluded patients in Supplementary Table 1.

**Table 1:** Demographic data of the total cohort and cohort with all measurements available required for modelling

	Total cohort	Modelling cohort		
		All	Non-progressing	Progressing
*N*	100	78	39	39
Progressing (*N*)	50	39	0	39
Female (*N*)	78	64	32	32
Median age at onset, years [range, IQR]	31.0 [15.0–59.0, 24.0–38.0]	31.0 [15.0–59.0, 24.0–38.0]	29.0 [16.0–51.0, 23.0–34.0]	34.0 [15.0–59.0, 25.5–42.0]
Median disease duration at baseline, years [range, IQR]	6.2 [0.0–40.5, 3.1–11.8]	6.5 [0.0–30.3, 3.3–11.6]	5.1 [0.0–17.2, 2.3–8.6]	9.4 [0.5–30.3, 4.8–12.7]
Median EDSS at baseline [range, IQR]	2.0 [0.0–6.5, 1.5–3.0]	2.0 [0.0–6.5, 1.5–3.0]	1.5 [0.0–6.5, 0.5–2.0]	2.5 [1.0–6.0, 2.0–4.0]
Median clinical follow-up time (years) [range, IQR]	7.8 [3.6–18.7, 6.5–9.8]	7.7 [3.6–17.1, 6.2–9.4]	8.2 [4.2–17.1, 7.4–11.5]	6.8 [3.6–11.9, 5.8–8.4]
Ethnic origin—East Asian, *N*	3	3	2	1
Ethnic origin—White, *N*	88	67	32	35
Ethnic origin—Eurasian, *N*	1	1	0	1
Ethnic origin—sub-continental, *N*	3	2	2	0
Ethnic origin—middle eastern, *N*	5	5	3	2
Symptoms at onset—brainstem-cerebellum, *N*	22	14	8	6
Symptoms at onset—optic pathways, *N*	32	25	14	11
Symptoms at onset—spinal cord, *N*	40	32	13	19
Symptoms at onset—supratentorial, *N*	12	11	5	6

### MRI processing

T1 and FLAIR brain MRI data were processed using ico**brain ms** version 5.5, a FDA510k-cleared and Ce approved software for fully automated cross-sectional and longitudinal brain and lesion volume quantification. This software is FDA-cleared, available for use and has been incorporated into clinical practice and was specifically developed to accurately quantify multi-scanner brain MRI as encountered in real-world settings.^26,27^ Additional image-processing methodology is detailed in the Supplementary Materials.

Brain T1 and/or sagittal T2 spinal cord MRI were processed with icospine, a research pipeline for cross-sectional measurement of cord lesion volume (on spinal cord T2) and cross-sectional area (on brain T1 or spinal cord T2). The cord lesion segmentation and volumetrics were performed using the well-established Spinal Cord Toolbox^28,29^—a comprehensive, open-source set of command-line tools dedicated to the processing and analysis of spinal cord MRI data.^30^ The Spinal Cord Toolbox builds on previously-validated methods and includes algorithms to segment and register new imaging data, which we utilized with additional in-house morphological refinements. In cases where both brain and spinal MRI were available, spinal MRI was used as input for the icospine pipeline. See Fig. 1B for a visual representation of the MRI processing.

#### MRI quality control

All MRI scans underwent an automated quality control (QC), verifying appropriate field of view, resolution, tissue contrast and absence of major artefacts. For pairs of longitudinal brain scans, the automated QC also takes into account consistency of scan protocols and alignment. In addition, the longitudinal evolution of volumes over time was inspected for outliers. Scans or scan pairs flagged by auto QC or outlier analysis were then manually verified by an in-house QC expert. In case of suboptimal segmentation or alignment, the measurement was discarded.

The need for additional harmonization was assessed using Kolmogorov–Smirnov testing, comparing distributions of measurements coming from different scanners. No significant differences were found between scanners, and thus no further data harmonization was needed to correct for inter-scanner differences, given the absence of important distribution differences in the measures described below. Additional image-processing information is provided in the Supplementary Materials.

#### Lesion volume measurements

The lesion volume in the three compartments was measured based on the T2 (FLAIR) hyperintensity. Volumes in cerebrum (L_cereb._) and infratentorium (L_infr._) were calculated from the brain MRI and cervical lesion volume (L_cord_) from the spinal cord T2 MRI. The segmentation of cervical lesions obtained from icospine was further refined with manual corrections performed by two raters. Lesion volumes in subcompartments for use in the patient-specific visualizations were calculated for juxtacortical, deep white matter, periventricular, medulla, pons midbrain, cerebellum, high cervical and low cervical regions.

#### Parenchymal volume measurements

The parenchymal volume (PV) in the three compartments was measured with the volumes of cerebral (P_cereb._) and infratentorial (P_infr._) brain tissue normalized for head size calculated from the T1 brain MRI, and the mean upper cervical cord cross-sectional area (mucca) from the spinal cord MRI. If no spinal cord MRI was available for a time point, mucca was computed using T1 brain MRI after calibration (calibration based on quadratic fit of mucca from time points with both T1 brain and spinal cord MRI).

Lesion volumes, tissue volumes, mucca and LPF were transformed into percentile values to make their scales comparable, increasing interpretability. As no reference values yet exist for spinal cord lesion measurements, all percentiles were computed with respect to the study sample.

### Lesion parenchymal fraction (LPF) definition and calculation

We defined the lesion parenchymal fraction (LPF) as the ratio of the regional T2 lesion volume divided by the regional parenchymal volume, utilizing mucca for the cervical cord (see Fig. 1 inset). The LPF was calculated in the three main compartments (spinal cord, infratentorial, cerebral hemispheres) as follows:

- LPF in the spinal cord ($\mathrm{LP}F_{\;\mathrm{cord}}\;=\;\frac{L_{\mathrm{cord}}}{P_{\mathrm{cord}}}$): volume of lesions detected in the spinal cord divided by mucca.
- LPF in the infratentorial region ($\mathrm{LP}F_{\;\mathrm{infra}}\;=\;\frac{L_{\mathrm{infra}}}{P_{\mathrm{infr}.}}\;$): volume of lesions detected in the cerebellum and brainstem divided by infratentorial tissue parenchymal volume.
- LPF in the cerebrum ($\mathrm{LP}F_{\mathrm{cereb}.\;}\;=\;\frac{L_{\mathrm{cereb}.}}{P_{\mathrm{cereb}.}}$): volume of lesions detected in the cerebrum divided by the cerebrum parenchymal volume (here, calculated as the whole brain parenchymal volume minus the infratentorial tissue volume).

### Matching clinical and MRI results

For modelling purposes, time points require parenchymal and lesion measurements as well as clinical data, without missing values. As clinical, brain MRI and spinal cord MRI were not always recorded on identical dates, a merging strategy was applied utilizing defined time windows. All visit dates per patient (clinical and MRI) were considered. On dates with a missing MRI result, the value was filled by the nearest available result, up to 15 days prior or after the date of the missing value. Assumptions derived from the topographical model framework were applied to fill remaining missing values. For lesion volumes, we assumed that lesions, once formed, are essentially permanent, thus lesion volumes were forward-filled to the next available datapoint. For parenchymal volumes, we assumed that brain and spinal cord tissue does not grow appreciably during adulthood, thus parenchymal volumes were back-filled to the previous available datapoint. Finally, missing clinical measurements were filled by forward and backward filling up to max. 9 months prior or after available clinical visits.

### MRI-based model for decoding EDSS

After data processing and data matching, every time point was characterized by MRI features describing lesion and tissue volume of the three model compartments (cerebrum, infratentorium and the upper cervical cord), and by the measured EDSS score.

Our proposed MRI-based model for describing disability introduces LPF, combining lesion and tissue measurements:


(1) $$\mathrm{EDS}S_{\mathrm{LPF}}=a\;{\mathrm{LPF}}_{\mathrm{cereb}.}\;+\;b\;{\mathrm{LPF}}_{\mathrm{infra}.}\;+\;c\;{\mathrm{LPF}}_{\mathrm{cord}.\;}+\;K.\mathrm{ag}e_{0}.$$

To evaluate our model, we also compare it with other models considering separately lesion volume (LV) and/or parenchymal volumes (PV) to describe disability. All models are listed in Supplementary Table 2.

The reference model was defined as a linear combination of age, lesion volume percentiles and parenchymal tissue volume or area percentiles (LPV) in each of the three compartments, i.e.:


(2) $$\mathrm{EDS}S_{\mathrm{LPV}}\;\;=\;a_{1}\;L_{\mathrm{cereb}.}\;+\;a_{2}\;P_{\mathrm{cereb}.}\;+\;b_{1}\;L_{\mathrm{infr}.}\;+\;b_{2}\;P_{\mathrm{infr}.}\;+\;c_{1}\;L_{\mathrm{cord}}\;+\;c_{2}\;P_{\mathrm{cord}}\;+\;K.\;\mathrm{ag}e_{0}.$$

In which, $L_{x}$ denote local lesion volume percentiles, $P_{x}$ denote percentiles of local parenchymal tissue volume or area (x∈[cerebral,infratentorial,cervicalcord]), and a, b and c are the different weighting factors for each term. $\;\mathrm{ag}e_{0}$ is the age at the initial time point. Two variations were made on this model, either using only lesion volume measurements:


(3) $$\mathrm{EDS}S_{\mathrm{LV}}\;\;=\;a_{1}\;L_{\mathrm{cereb}.}\;+\;b_{1}\;L_{\mathrm{infr}.}\;+\;c_{1}\;L_{\mathrm{cord}}\;+\;K.\mathrm{ag}e_{0}$$

or only parenchymal/tissue volume measurements:


(4) $$\mathrm{EDS}S_{\mathrm{PV}}=a_{2}\;P_{\mathrm{cereb}.}+b_{2}\;P_{\mathrm{infr}.}+c_{2}\;P_{\mathrm{cord}}+K.\;\mathrm{ag}e_{0}.$$

All models were fitted to the data using leave-one-patient-out cross-validation, i.e. per patient, the data of all other patients were used to train/fit the model. The resulting models were then applied on time points of the omitted patient to obtain the estimated EDSS for that patient. Model performance was evaluated across all time points in terms of Pearson correlation and root mean squared error between measured and estimated EDSS. Additionally, to evaluate how well the relative changes between time points were matched between the measured and estimated EDSS, the mean absolute error without patient bias was calculated after subtracting the mean error/bias for all time points of a given patient.

For our LPF-based model, we evaluated the relative contribution of the three compartments (cervical, infratentorial, cerebral). To do so, for each time point for which model fitting was done, we stored the resulting coefficients for each compartment (e.g. for the LPF model, the coefficients *a*, *b* and *c*). Descriptive statistics of the compartmental coefficients were then computed, and a density plot of their distributions was generated.

### Patient-specific visualization

EDSS and LPF longitudinal trajectories are visualized per individual patient to explicitly evaluate the topographical model representation. The matplotlib library in Python was used to render patient-specific EDSS and LPF longitudinal trajectories in four panels. The panels share the *x*-axis, representing the age of the patient and time period under investigation, and plot on their *y*-axes: bottom panel—lesion volume (ml); second panel from bottom—parenchymal volume (percentile); third panel from bottom—LPF (cumulative percentile); and top panel: EDSS and component FSS.

## Results

### Cohort overview

After MRI quality control and clinical-MRI time window matching, MRI data from 78 patients were sufficient for modelling, including 39 non-progressing and 39 progressing patients from RRMS to SPMS (a flowchart for the sample size is provided in Supplementary Fig. 1 including explication of excluded patients who did not have sufficient imaging data after quality control for the modelling calculations; a distribution of variables is described in Supplementary Table 3).

### MRI-based model ranking for decoding EDSS

The LPF model had the best performance in terms of low RMSE (1.638) and was ranked second in Pearson correlation (0.275) and mean absolute error without patient bias (0.518). The highest Pearson correlation was obtained with LPV (0.279), and the lowest mean absolute error without patient bias was found with the PV model (0.486). Performance of all models and all evaluation metrics are described in Supplementary Table 4.

### Contributions of compartments

As the models were fitted on a leave-one-patient-out cross-validation basis, we obtain per coefficient a distribution with *N* values (*N* = number of patients). For the LPF and LV models, as well as the lesion volumes in the LPV model, the cervical compartment had the largest mean coefficient, followed by the infratentorial and cerebral compartments, in that order.

**Figure 2:**
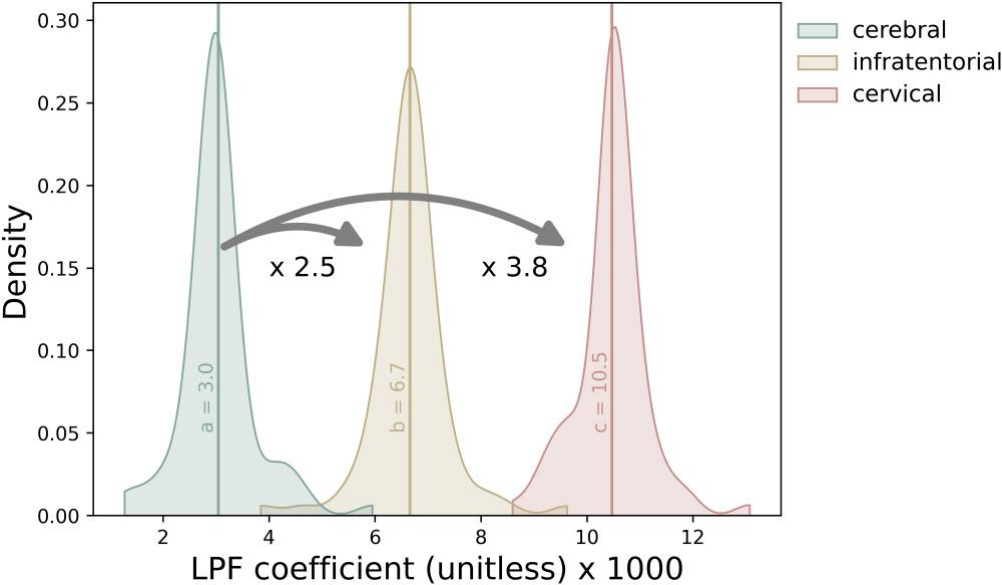
**Relative coefficients of the three topographical compartments.** Distribution of compartmental LPF coefficients, illustrating the relative importance of the cervical, infratentorial and cerebral compartments in describing disability. The *x*-axis represents the value of the coefficients (a, b and c in Supplementary Table 2). The *y*-axis density reflects the number of time points in which a certain coefficient value resulted from modelling. These results demonstrate that lesions in the cervical compartment contribute most in explaining disability, followed by the infratentorial compartment.

Focusing on the LPF model for estimating disability, Fig. 2 illustrates the distribution of compartmental coefficients. Setting the mean coefficient of cerebral LPF to 1, the cervical compartment had the largest coefficient (3.8), followed by infratentorial (2.5). Using these coefficient values, compartment-weighted cumulative LPF values depict MS disease trajectory longitudinally on a per-patient basis. The descriptive statistics of the LPF model coefficients, as well as for the other models, are listed in Supplementary Table 5.

### Case visualizations of the LPF model

**Figure 3:**
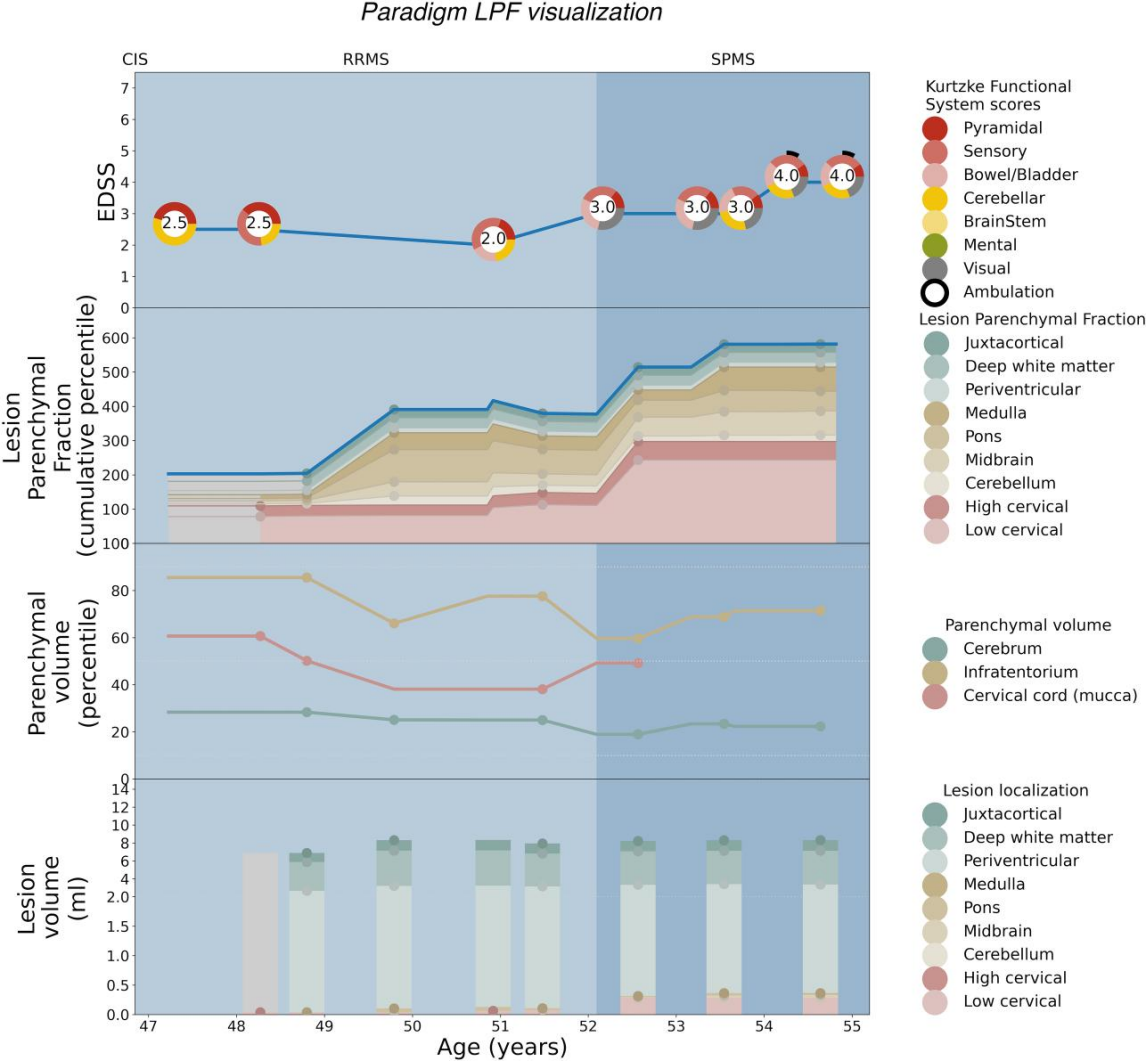
**Single-patient visualization of disease course over time using lesional and parenchymal metrics, lesion parenchymal fraction (LPF) and clinical trajectory.** Paradigm single-patient case depicted using the LPF technique. The *x*-axis shows the age range for the individual patient depicted. In the key at right for each panel, the lesion, parenchymal volume, LPF and clinical disability scores are shown, all colour-coded to denote topographical model compartment localization. Read from the bottom up, the bottom panel represents lesion volume depicted as a stacked bar graph at each MRI time point, divided as per the topographical model by colour into cervical cord lesions (shades of red), infratentorial lesions (shades of yellow) and cerebral hemisphere lesions (shades of green) graphed in ml on the *y*-axis. To allow the same range in the *y*-axis for patients with high or low lesion load, the *y*-axis range is split in two, with the bottom half covering the range between 0 and 2 ml and the upper half covering the range between 2 and 14 ml. To emphasize that the lesion occurrences are punctual events (rather than continuous), the stacked bar graphs are only shown for time points at which at least one (not forward or backward filled) MRI is available. Second panel from the bottom, parenchymal volume is shown as a line graph with percentile) shown on the *y*-axis, with parenchymal volume curves colour-coded to depict spinal cord volume derived from mean upper cervical cord area (mucca) shown as a red line; infratentorial volume (yellow line); and cerebral volume (green line), with measurement time points denoted with colour-coded dots. Third panel from the bottom, the lesion parenchymal fraction (LPF), combining lesion and parenchymal volume into a single metric, is depicted as a stacked graph of the three main LPF trajectories over time. The *y*-axis measures the cumulative percentile of LPF. The top-line LPF trajectory (blue line) is the weighted sum of the component LPF percentiles in the three main compartments, the weights being the relative coefficients of each compartment (i.e. 1 for cerebral LPF, 2.5 for infratentorial LPF and 3.8 for cervical LPF). The stacked LPF trajectories are furthermore coloured according to their constituting subcompartments, i.e. cervical cord LPF (shades of red); infratentorial LPF (shades of yellow); and cerebral hemisphere LPF (shades of green). Measurements are forward and backward filled, where actual measurements are indicated by dots. Areas for which no measurements are available before backward-/forward-filling (for instance, the absence of cervical cord imaging at a particular time point) are coloured grey to indicate missing data. The top panel depicts the clinical disability trajectory (blue line), measured on the *y*-axis as the EDSS score. EDSS is shown with the functional system scores (FSSs) depicted as normalized colour wheels to demonstrate both overall disability and individual clinical symptoms. Functional systems are categorized with the pyramidal, sensory and bowel and bladder symptoms referable to the spinal cord (shades of red); brainstem and cerebellar disease referable to the infratentorial compartment (shades of yellow); and the mental functional system score referable to the cerebral hemispheres (in green). Ambulatory dysfunction—which may be driven by deficits in multiple functional systems—is shown as a partial black circle extending clockwise around the colour wheel at time points where gait is affected. The visual functional system, referable to optic neuritis, does not have an MRI correlate and is shown in grey. The relative concordance of the cumulative top-line LPF trajectory (blue line), and that of the EDSS (blue line), can be visually inspected. At the top of the figure, clinician-determined phenotype is shown, including CIS, RRMS and SPMS as applicable; the figure background changes from light to dark blue at the time of conversion to SPMS. In this paradigm example, the patient developed increased disability during the 8 years of observation, converting from RRMS to SPMS at age 52. Mild disability is already seen at age 47 with cerebellar, sensory and pyramidal functional system involvement at EDSS 2.5, with worsening at the age of 52 with bowel/bladder impairment and visual symptoms at time of SPMS conversion at EDSS 3.0 (top panel). LPF is seen to increase in advance of clinical progression, with increase in cervical cord and infratentorial lesion burden and modest regional atrophy, such that the LPF profile follows a similar increasing pattern as the EDSS.

**Figure 4:**
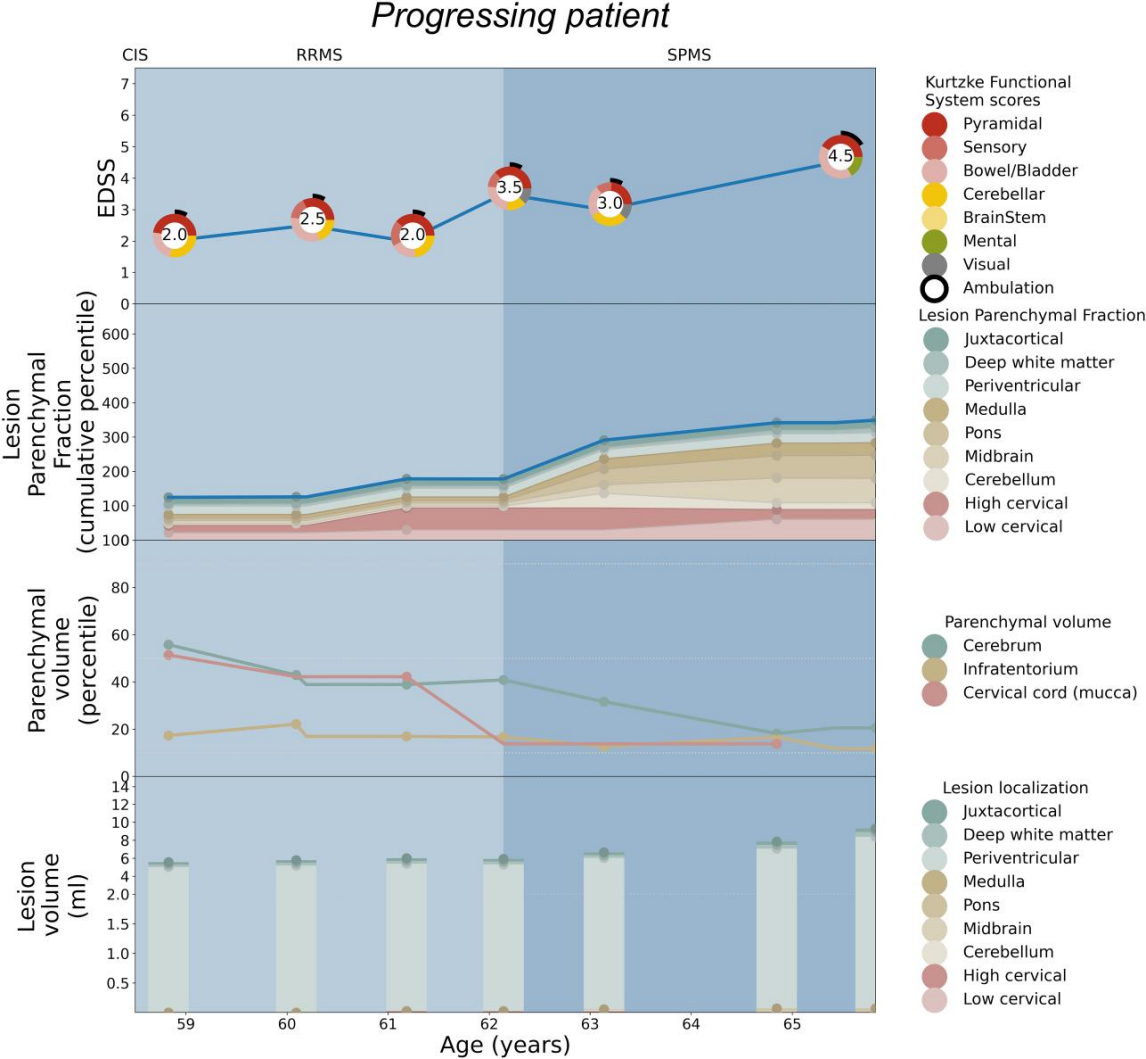
**Patient depiction demonstrating conversion from RRMS to SPMS and the corresponding LPF trajectories aligning with cervical and infratentorial involvement.** See the legend of Fig. 3 for a general explanation of the figure panels. The patient exhibits mild disability at the age of 58 with an EDSS of 2.0, continuously progressing to moderate disability and conversion to SPMS after the age of 62. At each time point, there were findings of pyramidal, sensory and cerebellar function impairment. Lesion burden only slightly increased, however the infratentorial parenchymal volume percentile was low from the outset, with cervical cord percentile reducing over time. The resulting cumulative LPF trajectory closely follows the continuously increasing disability curve, with symptoms predominantly driven by cervical and infratentorial compartments, matching the functional system scores.

**Figure 5:**
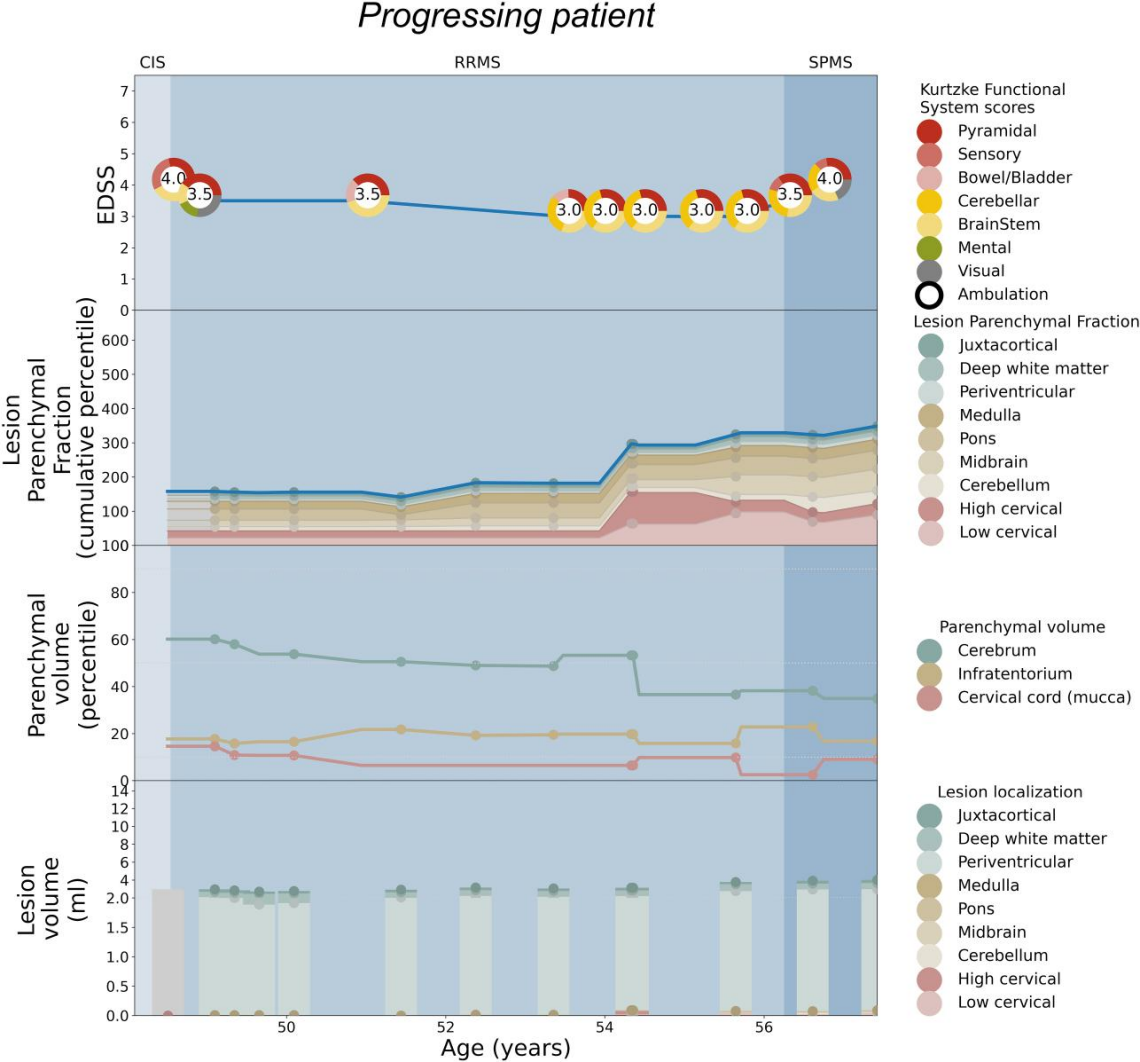
**Patient depiction demonstrating conversion from RRMS to SPMS and the corresponding LPF trajectories aligning with pyramidal involvement.** See the legend of Fig. 3 for a general explanation of the figure panels. This patient displays a pattern of increasing disability during 10 years of observation—although with some variability in the EDSS score, the trajectory towards increased disability (and conversion to SPMS at age 56) corresponding with the increase in cumulative LPF percentile. Of note is the increase in cervical cord lesion volume at age 54, that while small in volume, in combination with low cervical cord tissue percentile, this is visibly translated into a substantially increased cord LPF at the time of SPMS conversion. Approximately two years later at age 56, the patient’s disability trajectory worsens, in concordance with the cumulative LPF increase.

**Figure 6:**
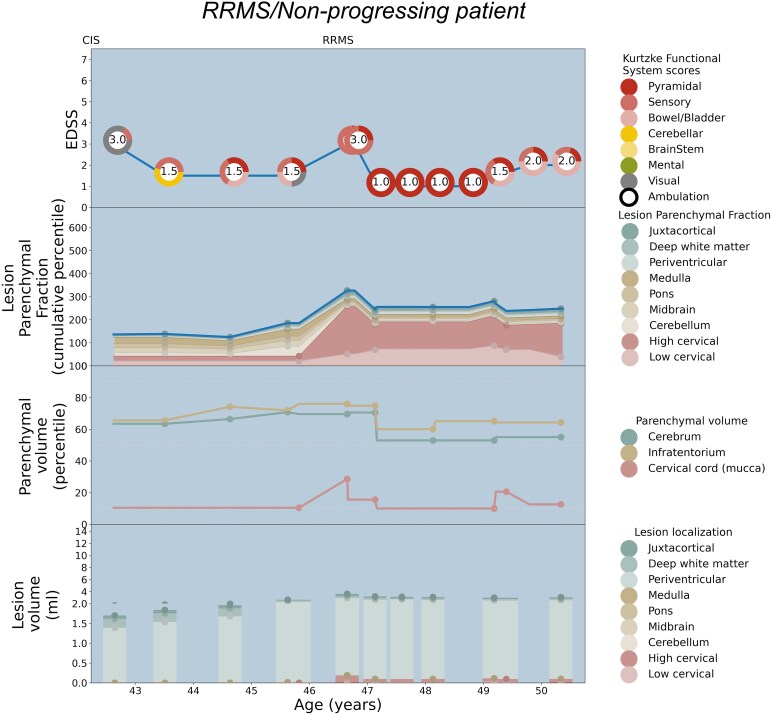
**Patient depiction demonstrating stable RRMS without conversion to SPMS, and the corresponding LPF trajectories aligning with pyramidal involvement.** See the legend of Fig. 3 for a general explanation of the figure panels. The patient demonstrates fluctuating disability without progressive accumulation, and thus is considered to remain RRMS during the 8 years of observation. Visual symptoms at the time of CIS at age 42 are shown in the top panel in grey on the colour wheel, as there is not a compartmental MRI correlate of optic neuritis. Mild disability at EDSS 1.5 with sensory and cerebellar signs is observed at age 43, corresponding to the mild LPF profile at this stage. Around age 47, worsening of pyramidal signs raises the EDSS to 3.0; an increase in cervical lesion burden with low cord parenchymal volume yields a corresponding acute increase in LPF at this time point. In the subsequent years, residual pyramidal tract signs remain along with concomitant increased plateau in the cervical cord LPF. The cumulative LPF trajectory tracks closely with the EDSS curve throughout the observation period. Interestingly, the cerebral and infratentorial tissue percentiles remained sufficiently high to avoid cerebellar or cerebral functional system disability, consistent with the role of compensatory reserve.

**Figure 7:**
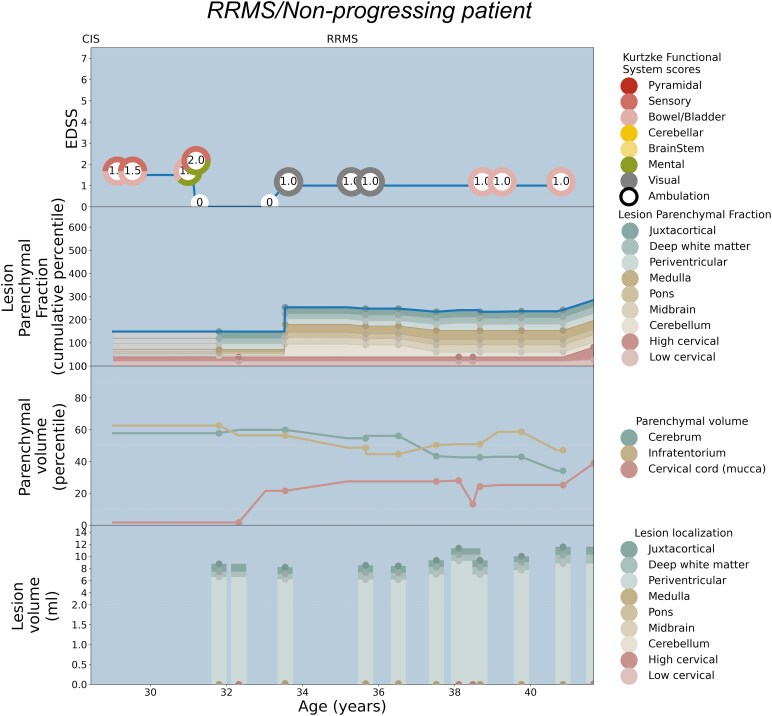
**Patient depiction demonstrating stable RRMS without conversion to SPMS, and the corresponding LPF trajectories aligning with minimal symptoms.** See the legend of Fig. 3 for a general explanation of the figure panels. The patient maintains mild disability between the ages of 28 and 42, with a period of time with an EDSS of 0 in her early 30s, and does not convert to an SPMS phenotype. Although she demonstrates a relatively large total lesion load (bottom panel), the lesions are mostly located in the cerebral hemispheres, which contribute least in this model to the overall EDSS score. The lesion volume in the spinal cord and infratentorial compartment is minimal, yielding a largely flat cumulative LPF profile consistent with the low disability curve. At several time points in her mid-30s, the patient’s EDSS score is 1.0 based on visual findings alone, shown in grey in the colour wheel as there is no MRI correlate for this functional system.

**Figure 8:**
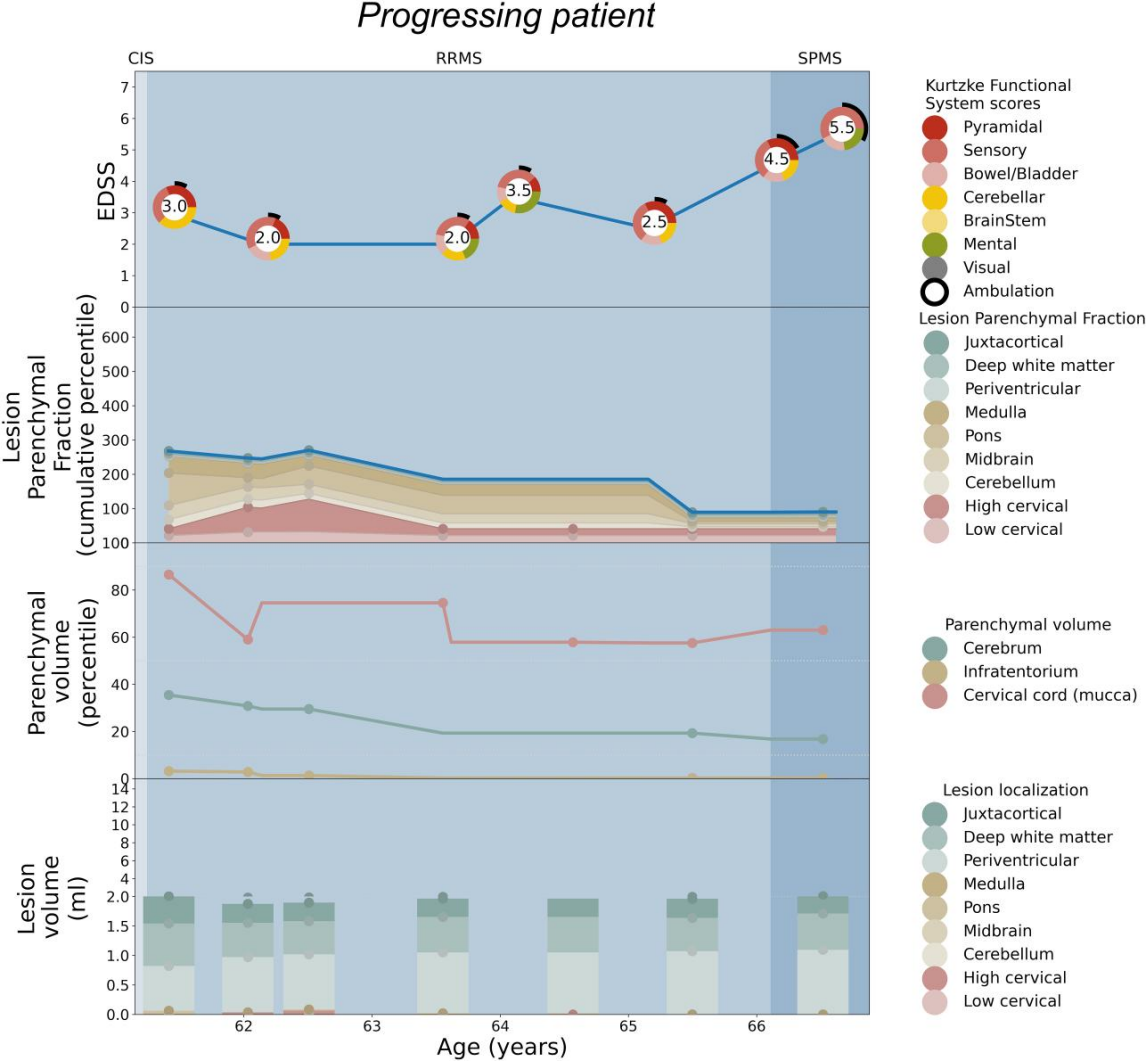
**Individual patient outlier, demonstrating a worse clinical course compared to the predicted disability trajectory based on LPF.** See the legend of Fig. 3 for a general explanation of the figure panels. This patient’s disability trajectory is worse than would be predicted by the LPF model. She demonstrated fluctuating disability status between the ages of 61 and 65, with findings in this time window related to cerebellar and pyramidal tract dysfunction as reflected in the cumulative LPF, largely driven by infratentorial lesions in combination with low regional parenchymal volumes. After the age of 65, however, the patient developed an upward disability progression trajectory, with increase in multiple functional system and ambulatory dysfunction, considered to have SPMS at age 66. The clinical progression was not reflected in the LPF profile, which remains low and decreases over time. This discordance may be explained by undersegmentation of infratentorial lesions or other unmeasured lesions, e.g. in the thoracic spinal cord, which are not part of the LPF trajectory as depicted here.

**Figure 9:**
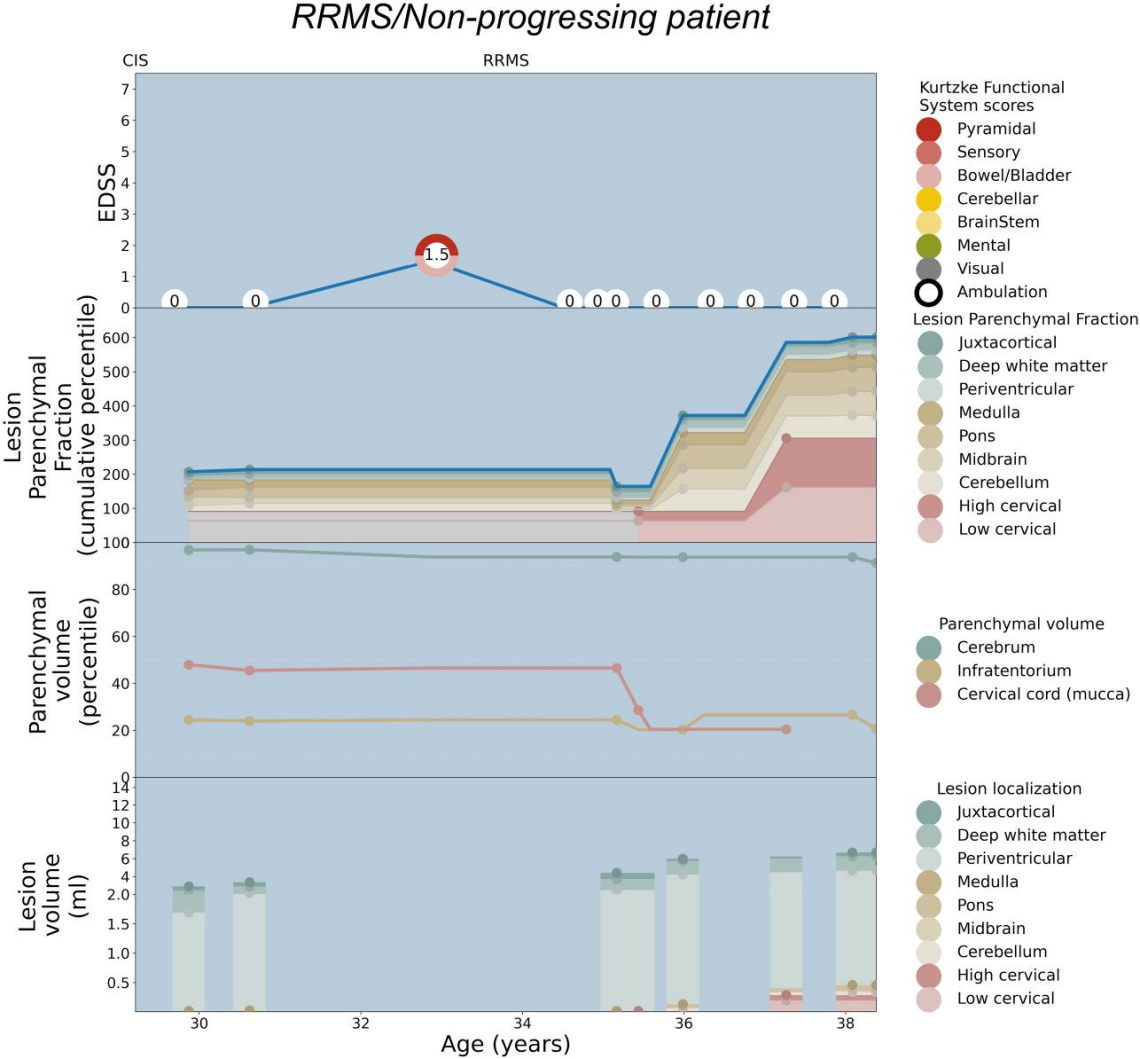
**Individual patient outlier, demonstrating a more favourable clinical course compared to the predicted disability trajectory based on LPF.** See the legend of Fig. 3 for a general explanation of the figure panels. This patient has a disability trajectory that does not track with the cumulative LPF measure of disease burden, with a better clinical course than would be predicted by the LPF model. She demonstrates minimal to no overt disability throughout the follow-up time over 9 years. While the initial LPF percentile is commensurately low (noting the absence of cord data during this period depicted in grey) at the age of 36, the LPF starts to increase, initially driven by infratentorial lesions, and at the age of 37 also by emerging spinal cord lesion load and rising cord LPF. This did not translate into increased disability, with the EDSS remaining 0 at age 38. While this could be attributed to MRI measurement error—where the model causes small changes in cord lesion or parenchymal volume to have an outside effect on cumulative LPF—it is possible that the high brain parenchymal volume (as seen in the second panel from the bottom) is indicative of a higher level of reserve sufficient to compensate for the lesion burden.

Patient-specific EDSS and LPF longitudinal trajectories were rendered utilizing the weighted coefficients for each topographical compartment as depicted in Fig. 2. Seven illustrative cases are shown: Fig. 3 serves as a paradigm case visualization; the legend details the information conveyed in each of the four panels. Figures 4 and 5 comprise two progressing patients who transitioned from the RRMS to SPMS phenotypes, and Figs 6 and 7 show two non-progressing patients who remain in the RRMS phenotype throughout the observation period. Figures 8 and 9 show two outlier cases where the LPF model does not approximate the EDSS trajectory; implications of the outliers are instructive as to the limitations of the current model.

## Discussion

The topographical model of MS was proposed to depict individual clinical course paradigms through the visualization of above-threshold and below-threshold disease.^13,31^ The hypotheses contained in this model—that lesion localization can be mapped to clinical findings, that there is a clinical threshold, and this threshold declines as reserve is depleted—have the potential to better characterize heterogeneity of disease activity and progression, and the relationship between them. In this study, we developed and evaluated LPF as a quantified expression of the topographical model, a first effort to operationalize this model to depict individual disease courses.

LPF integrates parenchymal volume and lesion localization together into a single metric to better model disability accumulation. We found that LPF is a better descriptor of EDSS than models utilizing parenchymal volume and lesion volumes individually, which has been the traditional approach to utilizing MRI metrics as prognostic indicators in MS. The fact that models including LPF outperformed those without it suggests that this metric may provide a way of extracting additional prognostic information from MRI scans using sequences obtained in routine clinical practice. The LPF approach can thus be utilized by other investigators to characterize disease burden in existing datasets where lesion burden and parenchymal volumes have been acquired.

We determined the relative coefficients of LPF for each of the three principal compartments towards estimating EDSS, finding that when setting the coefficient of the LPF in the cerebral hemispheres to 1, LPF in the cervical cord was 3.8 time more impactful, and the infratentorial compartment was 2.5 times more impactful. That LPF from the spinal cord and infratentorial regions conferred several-fold more impact on EDSS than that of the cerebral hemispheres is congruent with prior studies that found prognostic relevance of spinal cord^3-7^ and brainstem lesions,^2^ and emphasizes the importance of lesion localization along the caudal axis.^9^ One of the key elements of the topographical model is the interaction between the presence of lesions in a certain CNS region, the volume loss of that associated region, and the relationship with clinical symptoms. Our LPF findings validate the topographical model’s conceptualization of varying amounts of reserve in these three compartments, with the spinal cord at the shallow end of the reserve pool. This aligns with previous work^15,16^ showing that cervical cord atrophy is predictive of progressive disability accumulation; our study builds on this by incorporating the cord lesion volume measure directly into the regional LPF metric. Additionally, our results further refine the topographical model framework, in that while the original model depicts a single clinical threshold descending uniformly across anatomical regions in the CNS, we have now evaluated and depicted regional parenchymal volumes, moving at different rates, providing evidence of variability in progression rate in different anatomical compartments.

Using these weighted LPF values generated from the entire cohort, we generated novel visualizations of MS disease burden depicting cumulative LPF and clinical course measured by EDSS and its component functional systems longitudinally on a per-patient basis. These visualizations depict the extent to which the trajectory of LPF over time matches the severity and clinical pattern of disability for each patient. In representative cases where disability accumulated substantially and the patient was determined to have transitioned from RRMS to SPMS, the top-line cumulative LPF trajectory can be seen to track upwards adhering to that of the EDSS score. In cases where disability did not substantially increase and the patients were deemed to remain in the RRMS phenotype, the top-line cumulative LPF tracing often remained at a low percentile. Congruent with the evolution towards more personalized characterizations of disease course, these individualized disease course visualizations using the LPF technique yield insight into the pattern of disability accumulation over time, irrespective of phenotypic category, based on a patient’s specific pattern of lesion location and parenchymal volume loss—their individual ‘disease topography’. Utilizing the regional LPF metric may allow for greater clinical-MRI concordance to be derived from conventional MRI, and to help resolve the traditional clinical-MRI paradox.^13^

### Limitations

Although this work represents a useful step in translating the principles of the topographical model of MS into an MRI metric that can be empirically tested, we also showed two representative outliers whose clinical picture as assessed by EDSS departs from the expected trajectory predicted by the LPF model. These examples highlight limitations of the current model and suggest insights for additional research. In Fig. 8, the patient’s disability trajectory is worse than would be predicted by the LPF model, with accumulation of ambulatory dysfunction and an increase in EDSS without a corresponding rise in LPF. There are methodological challenges to lesion segmentation and quantification in the infratentorial and spinal cord compartments, and subtle lesions may have been missed or under-segmented, leading to an LPF underestimate. Our dataset did not include the thoracic spinal cord, so the current model may under-measure prognostically meaningful lesion burden in this compartment. This LPF underestimate is magnified in importance given the highly-weighted coefficients of the spinal cord LPF in the model.

Disability accumulation may also stem from factors not currently included in the model, including measures of lesion severity/heterogeneity in chronic active lesions^32,33^ meningeal infiltration^34^ and axonal attrition in the lesion core. Slow lesion expansion accounts for the development of progression observed in some cohort studies,^35^ however sequences needed to assess such lesional change, such as slowly expanding lesions and phase rim lesion, were not acquired in clinical practice during the observation period. Finally, other important non-imaging prognostic factors related to functional reserve outside our dataset such as comorbidities, smoking status, depression and other background factors (social determinants of health, educational attainment) that contribute to clinical outcomes could help to explain clinical outliers from our model.

In Fig. 9, despite a substantial increase in LPF, the patient’s disability trajectory remains flat with an EDSS of 0. This patient maintains a high-percentile brain volume throughout the observed disease course, suggesting that compensatory reserve may have been sufficient to compensate in a way not currently factored in our regional LPF model. Furthermore, there is likely to be more to compensatory reserve than parenchymal volume alone, which is an ongoing area of investigation.^36^ Additionally, it may be that the increase in LPF seen in this case is a harbinger of future disability not yet observed, and that the EDSS is not always sensitive enough to be an early indicator of the subthreshold burden of disease.^13^

These two outlier cases reflect limitations to our current study that can be considered at several levels. From an imaging perspective, an ongoing challenge remains that the most prognostically important localization in the spinal cord is where current imaging is least sensitive and prone to artefact.^13^ Inherent noise in the imaging data may have an outsized impact particularly in the infratentorial compartment and spinal cord, and as STIR sequences and axial scans are more sensitive for cord lesions than the T2 sagittal sequences utilized here, this could contribute to an underestimate of cord LPF. Compounding the issue of relatively low resolution of spinal cord imaging is our modest sample size with limited number of cervical cord scans for analysis. Ideally, the percentile representations used in this work would be derived from a large control population of patients with MS,^37,38^ however such a large database of volumetric and lesion measurements in the spinal cord is not yet available.

Our use of cohort-derived percentiles allowed us to rank-order the severity of disease, however the small sample size limits the granularity of our LPF percentiles, and could cause both over- and under-estimate of disease severity. Because of the relatively limited sample size in this work, we presented the results of a leave-one-out cross-validation, which allowed us to reach one of our main goals, i.e. to quantify the relative importance of each of the three compartments in the topographical model. Replicating this work on an independent test dataset to develop more refined LPF distributions outside of this cohort will be an important next step to establishing population norms for LPF. Noting that we developed lesion parenchymal fraction using a dataset that was built on the Lorscheider definition of SPMS, future work with other cohorts can explore how this approach can be applied to modelling progression independent of relapse activity (PIRA), which may allow additional nuance.^39^ The fact that the LPF metric can be calculated from conventional MRI scans offers many opportunities to extend this work using existing MS datasets.

### Future directions

Although LPF as evaluated in this project utilizes two essential measures of MS disease burden—T2 lesions and parenchymal volumes—additional imaging parameters may improve the accuracy of the model. The LPF numerator, lesion severity, is estimated here solely as a function of lesion size, and the assumption of lesion constancy (and the forward-filling of missing data) may not capture subtle lesion dynamics and progressive evolution within lesions, such as slowly enlarging lesions, cavitation and de- and remyelination,^40^ which certainly impact disease progression. Additional work could refine the LPF model by including other measures of lesion severity including e.g. T1 black holes, microstructural diffusivity characteristics, myelin water fraction and mapping lesion location not just to anatomical region but to specific white matter tracts of consequence using tractography. An additional element that remains to be addressed is that multiple lesions in a particular pathway—for instance, the pyramidal tract—may have prognostic implications beyond what we have assessed using overall LPF; tractography could enhance the model by taking this into account. The LPF denominator, parenchymal volume, can also be further refined to take into account segmental atrophy in domains that have been shown to be MS-specific and prognostically meaningful, such as cortical grey matter and thalamic volumes.^41,42^ Additionally, as reserve is dynamic and structural MRI metrics alone may not fully encapsulate it, measures of altered connectivity patterns using fMRI may yield insight into the disconnection patterns underlying clinical progression in MS. Cervical cord volume is an essential driver of LPF in this model, and methodologies to measure this continue to improve, including segmenting cord atrophy into white matter and grey matter compartments.^17^

Clinically, this study utilized EDSS and its functional systems as the outcome measure of disability. As mentioned, EDSS may not be a sufficiently sensitive measure of disease burden, and in particular the cognitive domain is largely overlooked.^43^ The EDSS is operator dependent, has subjective variability in its lower range and maxes out in higher range where it is essentially reduced to a mobility scale, limiting the conclusions of this study and others that rely upon it. In addition to larger sample sizes and additional imaging metrics, future studies of LPF could include more detailed cognitive and functional performance measures to add clinical nuance to the model. While we evaluated RRMS and SPMS patients in this cohort, application of this approach to early MS datasets could allow modelling and prediction of disability accumulation using PIRA as an outcome.

In conclusion, the LPF metric we have developed has shown potential as an MRI measure of disease course and severity that empirically validates principles of the topographical model. It presents a novel approach that will require further validation prior to be established as a biomarker of MS progression. Refinements to this exploratory work using larger populations and additional imaging metrics could allow application of this model to individual patients.

## Supplementary Material

fcaf280_Supplementary_Data

## Data Availability

Raw data were generated at the Brain and Mind Centre in the University of Sydney, Australia. Derived data supporting the findings of this study are available from the corresponding author upon reasonable request.
