# Supplementary material for: Depicting multiple sclerosis disease course using lesion parenchymal fraction: a quantified expression of the topographical model of multiple sclerosis
Source: Brain Commun. 2025 Jul 22;7(4):fcaf280. doi: 10.1093/braincomms/fcaf280 (PMC12308131; doi:10.1093/braincomms/fcaf280)
Supplement: fcaf280_Supplementary_Data [file fcaf280_supplementary_data.pdf]

Supplemental Material

Supplementary Methods

ico**brain ms**

Brain tissue volumes (white matter, (cortical) gray matter, lateral ventricle, hippocampus, thalamus) and lesion volumes (FLAIR hyperintensities, T1 hypo-intensities and Gd-enhanced T1 hyper-intensities) are quantified using ico**brain ms** software version 5.5.[1,2] The ico**brain ms** software consists of two steps: cross-sectional and longitudinal analysis.

Cross-sectional analysis

The cross-sectional analysis is a joint-analysis of T1 and FLAIR scan to segment the major tissue classes (gray matter (GM), white matter (WM) and cerebrospinal fluid (CSF)) as well as T1 and FLAIR lesions. It combines an unsupervised method with a supervised deep learning method. In the unsupervised part an expectation maximization algorithm optimizes a Gaussian mixture model on the image intensities while correcting for field inhomogeneities, guided by the probabilistic tissue priors. The algorithm includes a spatial consistency model based on a Markov Random Field (MRF) and iterates between the FLAIR lesion segmentation and tissue class segmentation of the lesion filled T1-weighted image until convergence. Lesion segmentations are then refined by a deep-learning attention-gate 3D U-net network. This deep-learning network is specifically trained to improve the segmentation of infratentorial and juxtacortical plaques. Thus, total lesion volumes are accurately quantified across the whole brain and per anatomical region according to the McDonald criteria (i.e. periventricular, infratentorial, juxtacortical or deep white matter).

From the main tissue classes, multiple tissue substructures are derived. Gray matter is further subdivided into cortical gray matter (CGM). Left and right thalamus are segmented on the T1-weighted image using a multi-atlas-based approach in combination with an intensity model, while also using the prior knowledge of the tissue class segmentations (WM, GM, CSF).

Longitudinal analysis

In a second step, the ico**brain ms** software evaluates longitudinal changes explicitly by jointly analyzing baseline and follow-up scans. As such, the software guarantees longitudinal consistency, important in case of scanner switches. Evaluation of global and local brain atrophy is performed using a registration-based approach, i.e. by performing Jacobian integration on the segmentations of each individual time point.

Changes in lesion volume are evaluated by performing a joint segmentation algorithm of the lesions of two subsequent scans, while explicitly quantifying the growing, shrinking and new lesions.
The longitudinal ico**brain** pipeline is evaluated on pairs of consecutive time points (having T1 scans with the same contrast) to compute the percentage volume change in tissues, ventricle and lesion types (in case both time points have FLAIR scans).[3]
For patients with more than two time points, the volumes per time point are computed by cumulating the volume changes that result from pair-wise longitudinal analyses.

Quality control and final sample size

For 28 out of 100 patients, the parenchymal volume measurements of one or more time points were discarded after quality control. This totaled to 39 out of 1390 analyzed brain MRI time points. For 13 of these time points, also lesion measurements were discarded. Because all patients had multiple time points, these discards did not directly lead to a reduced number of included patients. However, for modeling purposes it is required that all types of measurements (brain MRI, cord MRI, clinical) are available at nearby time points. Missing values of brain or cord MRI measurements were filled to 15 days prior and after the actual measurement date. Clinical data were matched up to 9 months from MRI imaging. Supplementary Figure 1 illustrates how the sample size available for modeling purposes was obtained.

Supplementary references

1. Jain S, Sima DM, Ribbens A, et al. Automatic segmentation and volumetry of multiple sclerosis brain lesions from MR images. *Neuroimage Clin*. 2015;8:367-375. doi:10.1016/j.nicl.2015.05.003

2. Rakić M, Vercruyssen S, Van Eyndhoven S, et al. icobrain ms 5.1: Combining unsupervised and supervised approaches for improving the detection of multiple sclerosis lesions. *Neuroimage Clin*. 2021;31:102707. doi:10.1016/j.nicl.2021.102707

3. Jain S, Ribbens A, Sima DM, et al. Two Time Point MS Lesion Segmentation in Brain MRI: An Expectation-Maximization Framework. *Front Neurosci*. 2016;10:576. doi:10.3389/fnins.2016.00576

Supplementary Figure 1. Flowchart illustrating the number of patients available for modeling starting from the initial patient count (N=100)

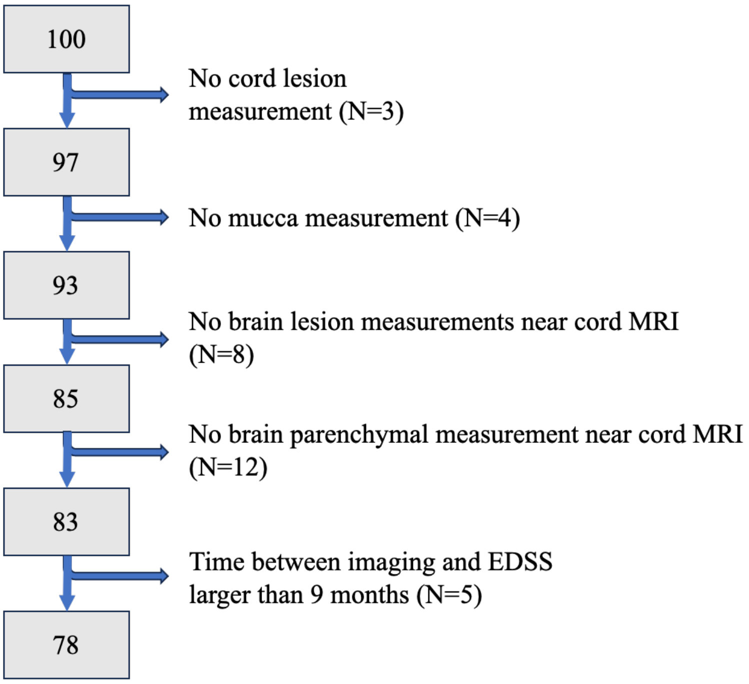

Supplementary Table 1. Demographic data of the total cohort, excluded patients, and cohort with all measurements available required for modeling.

	Total cohort				
			Modeling cohort		
	**Initial**	***Excluded***	**All**	**non-progressing**	**progressing**
N	100	*22*	78	39	39
progressing (N)	50	*11*	39	0	39
Female (N)	78	*14*	64	32	32
Median age at onset, years [range, IQR]	31.0 [15.0-59.0, 24.0-38.0]	*32.0 [15.0-51.0, 29.0-36.0]*	31.0 [15.0-59.0, 24.0-38.0]	29.0 [16.0-51.0, 23.0-34.0]	34.0 [15.0-59.0, 25.5-42.0]
Median disease duration at baseline, years [range, IQR]	6.2 [0.0-40.5, 3.1-11.8]	*3.9 [0.1-40.5, 0.8-11.9]*	6.5 [0.0-30.3, 3.3-11.6]	5.1 [0.0-17.2, 2.3-8.6]	9.4 [0.5-30.3, 4.8-12.7]
Median EDSS at baseline [range, IQR]	2.0 [0.0-6.5, 1.5-3.0]	*2.0 [0.0-5.0, 1.5-2.5]*	2.0 [0.0-6.5, 1.5-3.0]	1.5 [0.0-6.5, 0.5-2.0]	2.5 [1.0-6.0, 2.0-4.0]
Median clinical follow-up time (years) [range, IQR]	7.8 [3.6-18.7, 6.5-9.8]	*8.7 [5.3-18.7, 7.4-10.8]*	7.7 [3.6-17.1, 6.2-9.4]	8.2 [4.2-17.1, 7.4-11.5]	6.8 [3.6-11.9, 5.8-8.4]
Ethnic Origin - East Asian, N	3	*1*	3	2	1
Ethnic Origin - White, N	88	*21*	67	32	35
Ethnic Origin - Eurasian, N	1	*0*	1	0	1
Ethnic Origin - Sub-continental, N	3	*0*	2	2	0
Ethnic Origin - Middle eastern, N	5	*0*	5	3	2
Symptoms at Onset - Brainstem-Cerebellum, N	22	*8*	14	8	6
Symptoms at Onset - Optic Pathways , N	32	*7*	25	14	11
Symptoms at Onset - Spinal Cord , N	40	*8*	32	13	19
Symptoms at Onset - Supratentorial , N	12	*1*	11	5	6

Supplementary Table 2. List of linear models tested to estimate EDSS based on lesion and tissue volume form brain and spinal cord MRI. For the LPF model, coefficients a, b and c are visualized in main Figure 2.

**Model name**	**Model description**
**LPF**	$EDSS = a\, LPF_{cereb.} + b\, LPF_{infra.} + c\, LPF_{cord} + K.\, age_0$
**LV**	$EDSS = a_1\, L_{cereb.} + b_1\, L_{infr.} + c_1\, L_{cord} + K.\, age_0$
**PV**	$EDSS = a_2\, P_{cereb.} + b_2\, P_{infr.} + c_2 P_{cord} + K.\, age_0$
**LPV**	$EDSS = a_1\, L_{cereb.} + a_2\, P_{cereb.} + b_1\, L_{infr.} + b_2\, P_{infr.} + c_1\, L_{cord} + c_2 P_{cord} + K.\, age_0$

Supplementary Table 3. Cohort overview on the total sample and selected sample for modeling

	Total sample				
			Modeling sample		
	Initial	*Excluded*	All	non-progressing	progressing
N patients	100	*22*	78	39	39
Median number of brain MRI, N [range, IQR]	8.0 [2.0-17.0, 6.0-10.0]	*7.5 [2.0-13.0, 6.0-9.8]*	4.0 [1.0-13.0, 3.0-7.0]	4.0 [1.0-13.0, 2.5-8.0]	5.0 [1.0-11.0, 3.0-6.5]
Median brain MRI follow-up time, years [range, IQR]	7.1 [4.0-10.2, 5.9-8.2]	*7.4 [5.2-10.0, 6.6-8.4]*	4.2 [0.0-8.8, 1.8-5.9]	5.3 [0.0-8.8, 1.5-6.5]	3.9 [0.0-8.8, 2.0-5.3]
Median number of spinal MRI, N [range, IQR]	2.0 [1.0-10.0, 2.0-4.0]	*1.0 [1.0-3.0, 1.0-2.0]*	4.0 [1.0-13.0, 3.0-7.0]	4.0 [1.0-13.0, 2.5-8.0]	5.0 [1.0-11.0, 3.0-6.5]
Median spinal MRI follow-up time, years [range, IQR]	3.8 [0.0-10.2, 0.7-6.6]	*0.0 [0.0-8.9, 0.0-1.1]*	4.2 [0.0-8.8, 1.8-5.9]	5.3 [0.0-8.8, 1.5-6.5]	3.9 [0.0-8.8, 2.0-5.3]
**Whole brain at baseline, median [range, IQR]**					
WB tissue volume normalized, ml	1534.1 [1403.6-1717.2, 1495.2-1584.4]	/	1522.7 [1406.1-1718.7, 1487.4-1576.1]	1548.2 [1417.3-1718.7, 1519.6-1578.5]	1496.5 [1406.1-1657.3, 1482.0-1541.1]
WB lesion volume, ml	4.3 [0.7-24.7, 2.4-9.2]	/	4.9 [0.7-23.3, 2.5-9.4]	4.2 [0.7-23.3, 1.8-9.1]	5.4 [0.9-22.7, 2.7-11.0]
LPF, %	0.4 [0.1-2.1, 0.2-0.9]	/	0.5 [0.1-2.3, 0.2-0.9]	0.4 [0.1-2.1, 0.2-0.9]	0.5 [0.1-2.3, 0.3-1.0]
**Cerebrum at baseline, median [range, IQR]**					
Cerebral tissue volume normalized, ml	1340.6 [1210.8-1515.5, 1306.1-1388.1]	/	1327.5 [1204.5-1514.3, 1295.5-1369.5]	1343.7 [1218.6-1514.3, 1314.2-1380.3]	1303.5 [1204.5-1471.7, 1280.6-1350.8]
Cerebral lesion volume, ml	4.3 [0.7-24.6, 2.4-9.2]	/	4.9 [0.6-23.2, 2.4-9.2]	4.2 [0.6-23.2, 1.8-9.1]	5.4 [0.8-22.6, 2.6-10.9]
LPF, %	0.4 [0.1-2.4, 0.2-1.0]	/	0.5 [0.1-2.6, 0.3-1.0]	0.4 [0.1-2.5, 0.2-1.0]	0.5 [0.1-2.6, 0.3-1.1]
**Infratentorium at baseline, median [range, IQR]**					
Infratentorial tissue volume normalized, ml	196.6 [164.8-225.5, 187.2-203.5]	/	199.6 [170.3-233.9, 188.4-207.9]	204.5 [170.4-226.8, 194.9-211.6]	196.0 [170.3-233.9, 186.8-201.5]
Infratentorial lesion volume, ml	0.0 [0.0-0.6, 0.0-0.0]	/	0.0 [0.0-0.6, 0.0-0.1]	0.0 [0.0-0.3, 0.0-0.0]	0.0 [0.0-0.6, 0.0-0.1]

LPF, %	0.0 [0.0-0.4, 0.0-0.0]	/	0.0 [0.0-0.5, 0.0-0.0]	0.0 [0.0-0.2, 0.0-0.0]	0.0 [0.0-0.5, 0.0-0.0]
**Cervical measurements at baseline, median [range, IQR]**					
Mucca, mm^2	72.2 [44.0-89.0, 64.4-76.6]	/	71.4 [45.1-88.0, 65.4-74.6]	73.7 [59.7-88.0, 70.8-76.9]	66.3 [45.1-87.9, 63.7-73.3]
Cervical lesion volume, ml	0.1 [0.0-1.2, 0.0-0.2]	/	0.1 [0.0-1.0, 0.0-0.3]	0.1 [0.0-0.6, 0.0-0.2]	0.2 [0.0-1.0, 0.1-0.4]

Supplementary Table 4. Model performance based on different evaluation metrics: Pearson correlation and root mean squared error (RMSE)

Model	Pearson correlation	RMSE	Mean absolute error without patient bias
**LPF: Lesion Parenchymal Fraction**	0.275	**1.638**	0.518
LV: Lesion Volumes	0.267	1.642	0.520
LPV: Lesion and Parenchymal Volumes	**0.279**	1.650	0.523
PV: Parenchymal Volumes	0.192	1.702	**0.486**

Supplementary Table 5. Coefficients of models describing EDSS based on age and percentiles of compartmental MRI features.

Values are summarized across patients (N=78), and multiplied by 1000 for readability.

Model	Coefficient	mean	std	min	25%	50%	75%	max	cov
LPF	cerebral (a)	3.0	0.7	1.3	2.8	3.0	3.2	5.9	0.22
	infra (b)	6.7	0.7	3.8	6.5	6.7	6.9	9.6	0.11
	cervical (c)	10.5	0.6	8.6	10.3	10.5	10.7	13.1	0.06
	age (K)	40.7	0.8	36.7	40.5	40.7	40.9	42.8	0.02
LV	cerebral (a)	3.3	0.7	1.6	3.0	3.3	3.4	6.3	0.21
	infra (b)	6.0	0.8	3.1	5.8	6.0	6.2	9.3	0.13
	cervical (c)	11.0	0.7	9.2	10.9	11.0	11.3	13.5	0.06
	age (K)	41.4	0.8	37.5	41.2	41.4	41.6	43.2	0.02
PV	cerebral (a)	0.0	0.0	0.0	0.0	0.0	0.0	0.1	8.83
	infra (b)	3.0	0.5	1.7	2.7	3.0	3.2	4.9	0.18
	cervical (c)	1.4	0.5	0.0	1.1	1.4	1.6	3.0	0.39
	age (K)	62.2	0.8	59.3	61.9	62.2	62.6	65.4	0.01
LPV	Cerebral lesions (a1)	2.7	0.7	1.2	2.4	2.7	2.9	5.9	0.24
	Infratentorial lesions (b1)	5.8	0.8	2.9	5.6	5.8	6.0	8.8	0.13
	Cervical lesions (c1)	12.6	0.7	10.8	12.5	12.6	12.9	15.3	0.06
	Cerebral tissue (a2)	0.1	0.2	0.0	0.0	0.0	0.0	1.0	3.58
	Infratentorial tissue (b2)	6.2	0.6	4.5	5.9	6.2	6.4	9.0	0.09
	Cervical tissue (c2)	0.0	0.1	0.0	0.0	0.0	0.0	0.5	5.19
	Age (K)	47.0	0.8	44.3	46.7	47.0	47.3	49.9	0.02
